# Vitamin D status in healthy Italian school-age children: a single-center cross-sectional study

**DOI:** 10.1186/s13052-023-01422-x

**Published:** 2023-02-22

**Authors:** Tiziana Galeazzi, Sara Quattrini, Dorina Pjetraj, Simona Gatti, Chiara Monachesi, Elisa Franceschini, Luisita Marinelli, Giulia N. Catassi, Elena Lionetti, Carlo Catassi

**Affiliations:** 1 Department of Pediatrics, Marche Polytechnic University, 60123 Ancona, Italy; 2 Azienda Ospedaliero Universitaria delle Marche, 60123 Ancona, Italy; 3 Department of Pediatrics, University La Sapienza, 00185 Rome, Italy; 4 Center for Celiac Research, Mass General Hospital for Children, Boston, MA 02114 USA

**Keywords:** Vitamin D, 25(OH) D, School-age children, Prevalence, Deficiency, BMI, Season

## Abstract

**Background:**

Vitamin D is involved in calcium homeostasis and bone metabolism, although its extra-skeletal actions are also well-known. Low serum 25(OH)D levels are common both in adults and children worldwide.

**Methods:**

The purpose of this cross-sectional study was to determine the distribution of 25(OH)D levels in a cohort of healthy Italian school-age children, aged 5–10 years, in relationship to determinants of vitamin D deficiency such as season, BMI, gender, age and ethnicity.

**Results:**

The mean serum 25(OH) D level was 28.2 ng/mL; the prevalence of 25(OH)D sufficiency (> 30 ng/mL), insufficiency (20–30 ng/mL), deficiency (10–20 ng/mL) and severe deficiency (< 10 ng/mL) was 36%, 37%, 21% and 6% of the study-group population, respectively. The lower serum 25(OH)D values were observed during winter (21.6 ng/mL) and spring (22.9 ng/mL), as compared to summer (46.7 ng/mL) (*p* < 0.001). Higher BMI z-scores were associated with lower 25(OH)D level while no statistical difference was observed as related to gender and age groups.

**Conclusions:**

Healthy Italian schoolchildren show low 25(OH)D levels, particularly during winter and spring time. Seasonality, ethnicity and overweight/obesity were confirmed to influence the vitamin D status, thus indicating the need for effective initiatives to support adequate vitamin D status in this population group.

## Introduction

Vitamin D is an essential nutrient for maintenance of serum calcium and phosphorus homeostasis and its fundamental role in bone mineralization and bone mass acquisition during childhood is well-known [1]. Extra-skeletal actions of vitamin D are also established, suggesting a link between vitamin D deficiency and several chronic disorders, such as type 1 and type 2 diabetes, connective tissue disorders, inflammatory bowel disorders, chronic hepatitis, food allergies, celiac disease, asthma and respiratory infections, and cancer [2–5].

The vitamin D supply is both exogenous and endogenous, with the latter representing the principal source. The most notable dietary sources of vitamin D are fish liver oils (especially cod), fatty fish (i.e. salmon, herring), egg yolk, mushrooms and some dairy products (i.e. butter, whole and fortified milk, cheese and yogurt). The endogenous synthesis in the skin in response to ultraviolet B irradiation is the most notable source of Vitamin D, providing 90% of the total requirement, and it is highly variable depending on age, skin pigmentation and area of exposed skin, length and time of exposure and season [6–8].

The current Recommended Daily Allowance (RDA) in U.S. and Europe (i.e. between 400 and 600 IU/die, corresponding to 10–15 mcg/die) is readily achievable through casual sun exposure in the midday lunch hour at high latitudes and all the year at low latitudes [9]. Once it is produced in the skin or ingested from the diet, vitamin D is hydroxylated sequentially in liver and kidney to its biologically active form 1,25-dihydroxyvitamin D (1,25(OH)D). This hormone interacts with its receptors in the small intestine and in bone tissue to regulate calcium and phosphate absorption [10]. Vitamin D nutritional status is defined by the measurement of serum 25(OH)D concentrations, which is the major circulating vitamin D metabolite, and the most suitable indicator of vitamin D status with a half-life of 2–3 weeks [11, 12].

Although there are evidences for an increased risk of rickets with serum levels of 25-hydroxyvitamin D (25(OH)D) < 10 ng/mL [6, 13], there has been controversy about the optimal vitamin D levels required to maintain bone health, as well as the thresholds of this metabolite concentrations used to define deficiency or insufficiency. In the last years a number of agencies [German Nutrition Society, Dutch Health Ministry, Nordic Council of Ministers (NORDEN), UK Scientific Advisory Committee on Nutrition (SACN), European Food Safety Authority (EFSA), European Society for Paediatric Gastroenterology Hepatology and Nutrition (ESPGHAN)] [10, 14–18] revised the definition of vitamin D status, proposing different cut-off levels for sufficiency (> 20 ng/mL or ≥ 30 ng/mL), insufficiency (10–20 ng/mL or 20–30 ng/mL), deficiency (10–20 ng/mL or < 10 ng/mL), severe deficiency (< 10 ng/mL or < 5 ng/mL). In particular, in 2018 a consensus of the Italian Pediatric Society and the Italian Society of Preventive and Social Pediatrics (Società Italiana di Pediatria Preventiva e Sociale, SIPPS) suggested to define serum 25(OH)D threshold for sufficiency at ≥ 30 ng/mL and recommended vitamin D supplementation in all infants in the first year of life, independently of type of feeding [1].

Based on the vitamin D deficiency threshold of 20 ng/mL (severe if < 10 ng/mL), this is a global health problem, although the prevalence of low vitamin D status among children shows a high degree of variability across studies and countries [19, 20]. Epidemiological studies show a high prevalence of hypovitaminosis D (above 50%) throughout Italy with adolescents being particularly at risk [21, 22].

The aim of this study, conducted as a spin-off investigation from a mass screening study for celiac disease, was to assess the distribution of 25(OH)D levels in a cohort of healthy Italian school-age children living in the central area of Italy (Ancona, latitude 43°35′56"76 N), in order to clarify normal vitamin D levels in a sample of children belonging to the healthy general population.

## Materials and methods

### Study population

A sample of healthy Italian school-age children (5–10 years old), enrolled in a mass school screening for celiac disease study conducted in Ancona between 2015 and 2017, was considered eligible and offered to participate in this spin-off study [23]. Ancona is located in Central Italy at latitude 43° 35′ 39 N, and, on average, has around 2,220 sunshine hours per year. July is the sunniest month, and November has the lowest amount of sunshine. Diseases known to affect vitamin D metabolism, and vitamin D supplementation in the 12 months prior to the enrolment to the study were exclusion criteria. For each child, anthropometric and clinical data (age, gender, weight, height, BMI, ethnicity, residence, season of vitamin D blood sampling) were recorded.

Ethnicity was categorized as Caucasian or non-Caucasian (comprising African, Hispanic, and Asian). Growth parameters were collected during clinical evaluation before venous blood sampling by the same trained operator. Standing height was measured to the nearest 5 mm using a wall-mounted stadiometer (SECA 220, Germany); body weight was measured with a mechanical balance (SECA 200, Germany, 100 g of accuracy range). BMI was expressed as weight (kg)/height (m2) and reported also as Z-score. BMI categories were defined according to the World Health Organization (WHO) standards, as follows: underweight, normal, overweight, and obese (BMI < 5th, 5-85th, 85–95th, and ≥ 95th percentile, respectively) [24]. Residence was categorized as urban or rural.

The study was conducted in accordance with the principles of the Helsinki Declaration as revised in Fortaleza 2013 and the Institutional Review Board of the Polytechnic University of Marche (Ancona, Italy) approved this study protocol (protocol number 214592).

### Serum 25-hydroxyvitamin D determination

Total serum 25-hydroxyvitamin D (D2 + D3) level [25(OH)D] was quantitatively determined by chemiluminescent-immunoassay (CLIA) (LIAISON, DiaSorin, Italy), and expressed as ng/mL. According to the consensus of the SIPPS [1], the following cut-offs were considered to define the vitamin D status: sufficiency ≥ 30 ng/mL, insufficiency 20–29 ng/mL, deficiency < 20 ng/mL, severe deficiency < 10 ng/mL. The term hypovitaminosis D refers to serum 25(OH)D levels < 30 ng/mL. Serum calcium (Ca) and phosphate (P) levels were measured by absorbance using Dimension Vista® System for CA and PHOS (Siemens Healthcare Diagnostic, USA).

### Outcome measures

The primary outcome was the serum 25(OH)D level and the percentage of children with vitamin D insufficiency (< 30 ng/mL, according to SIPPS). Secondary outcome measures were: the correlation between serum 25(OH)D level and age; the percentage of children with vitamin D deficiency according to BMI, gender and ethnicity; the variation of 25(OH)D level during the four seasons; cumulative frequency distribution of serum 25(OH)D in our healthy Italian children population.

### Statistical analysis

Results are reported as mean ± standard deviation (SD), median (range) or percentages, as appropriate. The Student’s t-test (for normally distributed data) or the Mann–Whitney U-test (for non-normally distributed data) or the one-way analysis of variance (ANOVA) were used for comparisons. Differences in frequencies were evaluated with the χ2 test. A Multiple Regression Analysis was performed to assess the predictive value of presumed risk factors for hypovitaminosis D.

Statistical significance was set at *p* < 0.05. GraphPad Prism software (version 7, GraphPad Software, La Jolla, CA, USA), IBM SPSS Statistic v.23.0 (SPSS Inc, Chicago, Illinois, USA) and Microsoft EXCEL (v.2010; Microsoft Corp Redmond, Washington) were used for the analysis.

## Results

Out of the 1706 children serologically evaluated in the original mass screening study, 470 accepted to participate in this spin-off study. The characteristics of the study population are reported in Table 1.

**Table 1:** Characteristics of the study population

Variable	Total sample (*N* = 470)
Age (years – mean ± SD)	8.1 ± 1.2
Weight (kg – mean ± SD)	30.6 ± 11.8
Height (cm – mean ± SD)	129.5 ± 8.8
BMI (kg/m2 – mean ± SD)	17.9 ± 4.5
Z-score BMI (mean ± SD)	0.50 ± 1.1
BMI percentile (mean ± SD)	64 ± 29.6
Gender N (%)	
Male	247 (52.6)
Female	223 (47.4)
Ethnicity N (%)	
Caucasian	425 (90.4)
Non-caucasian	45 (9.6)
Season of blood sample N (%)	
Winter	167 (35.5)
Spring	114 (24.3)
Summer	111 (23.6)
Fall	78 (16.6)
[25(OH)D] (ng/mL; mean ± SD)	28.2 ± 9.4

The mean BMI was 17.9 kg/m2 (Z-score 0.504; 64th percentile) and the prevalence of underweight, normal weight, overweight and obesity was 3%, 62%, 20%, and 15%, respectively.

Overall, median 25(OH)D, Ca, P, and Ca/P serum levels were 26.2 ng/mL, 9.3 mg/dL, 4.9 mg/mL and 1.9, respectively; all these parameters yielded normal values in the study population.

**Fig. 1:**
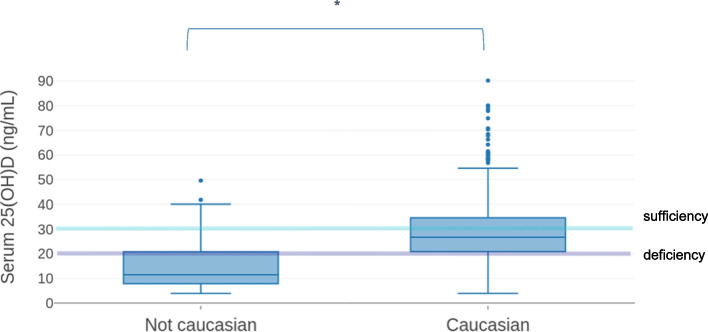
Serum 25-hydroxyvitamin D [25(OH)D] distribution according to ethnicity. The category “Non-Caucasian” comprises children of African, Hispanic, and Asian origins. Data are expressed as ng/mL. Median values are 11.5 ng/mL in the non-Caucasian subjects and 26.7 ng/mL in the Caucasian subjects. The Caucasian subjects show the highest levels of 25(OH)D (90.1 ng/mL vs 49.6 ng/mL), with the higher number of outliers. Significance p values (*p* < 0.005) are marked as (*)

**Fig. 2:**
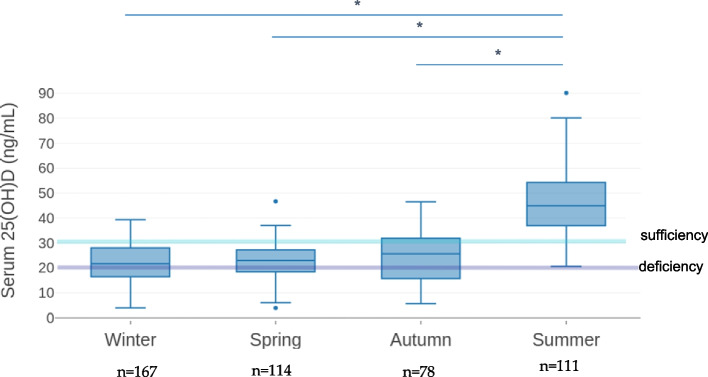
Seasonal fluctuation in the median serum 25-hydroxyvitamin D [25(OH)D] distribution. Data are expressed as ng/mL. Median values are 21.7 ng/mL in winter, 22.9 ng/mL in spring, 25.7 ng/mL in autumn and 44.9 ng/mL in summer. The lowest levels of 25(OH)D were registered in winter and spring with 3.9 ng/mL each. Summertime is the period with the highest median serum 25(OH)D level and interquartile range (IQR of 17.35); none of the participants presented a state of deficiency (25(OH)D < 20 ng/mL) during summer. Significance p values (*p* < 0.005) are marked as (*)

**Fig. 3:**
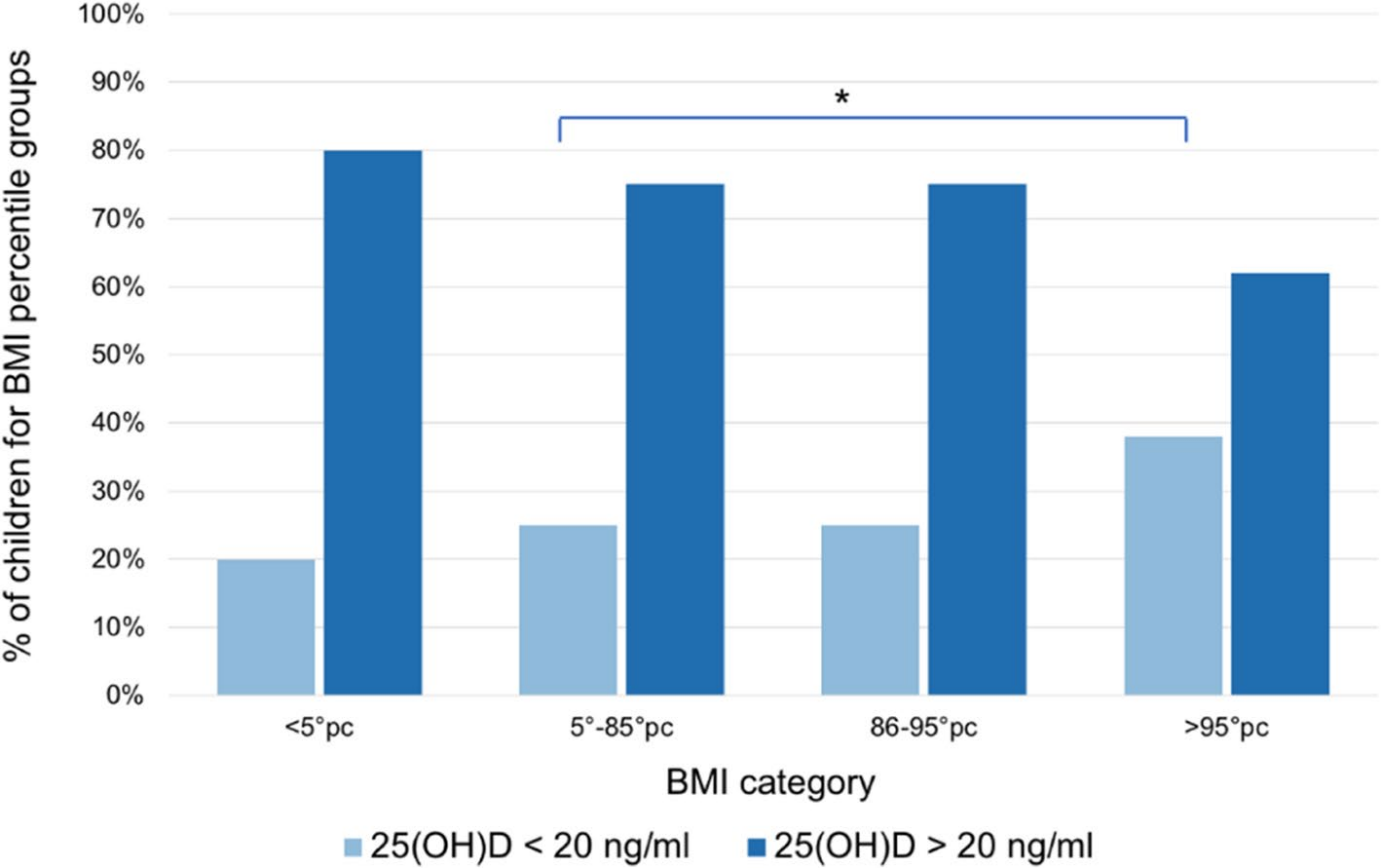
Percentage of children by serum 25-hydroxyvitamin D [25(OH)D] level under and over 20 ng/mL for BMI percentile groups. The obese group (BMI > 95 percentile) presents a significant higher prevalence of 25(OH)D deficiency when compared with the normal weight group (5–85 percentile). Significance p values (*p* < 0.005) are marked as (*)

Vitamin D level has been evaluated on the basis of ethnicity, season of blood sampling and BMI and is reported in Fig. 1, 2, 3, respectively. Median 25(OH)D level was significantly lower among non-Caucasian than in Caucasian children (*p* < 0.0001) (Fig. 1).

Serum 25(OH)D levels were found to be significantly higher in samples collected in summer, as compared to samples collected in winter, spring and fall (mean values: 46.7 vs. 21.6, 22.9 and 24.2, respectively; *p* < 0.0001), (Fig. 2).

No differences were observed in seasonal fluctuation between the two-ethnicity categories (data not shown). Non-Caucasian children showed lower vitamin D values than Caucasians, both as for median value of all seasons (as reported in Fig. 1), and as values referring to the each sampling seasons, but the seasonal trend remained similar for both groups.

**Fig. 4:**
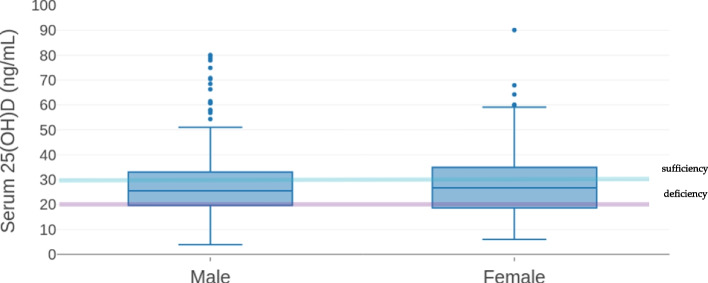
Serum 25-hydroxyvitamin D [25(OH)D] distribution according to gender. Median values are 25.5 ng/mL in males, 26.7 ng/mL in females. IQR is 13.4 ng/mL and 16.2 ng/mL in males and females respectively. Minimum and maximum 25(OH)D level are 3.9 ng/mL and 80.1 ng/mL in males and 6 ng/mL and 90.1 ng/mL in females

**Fig. 5:**
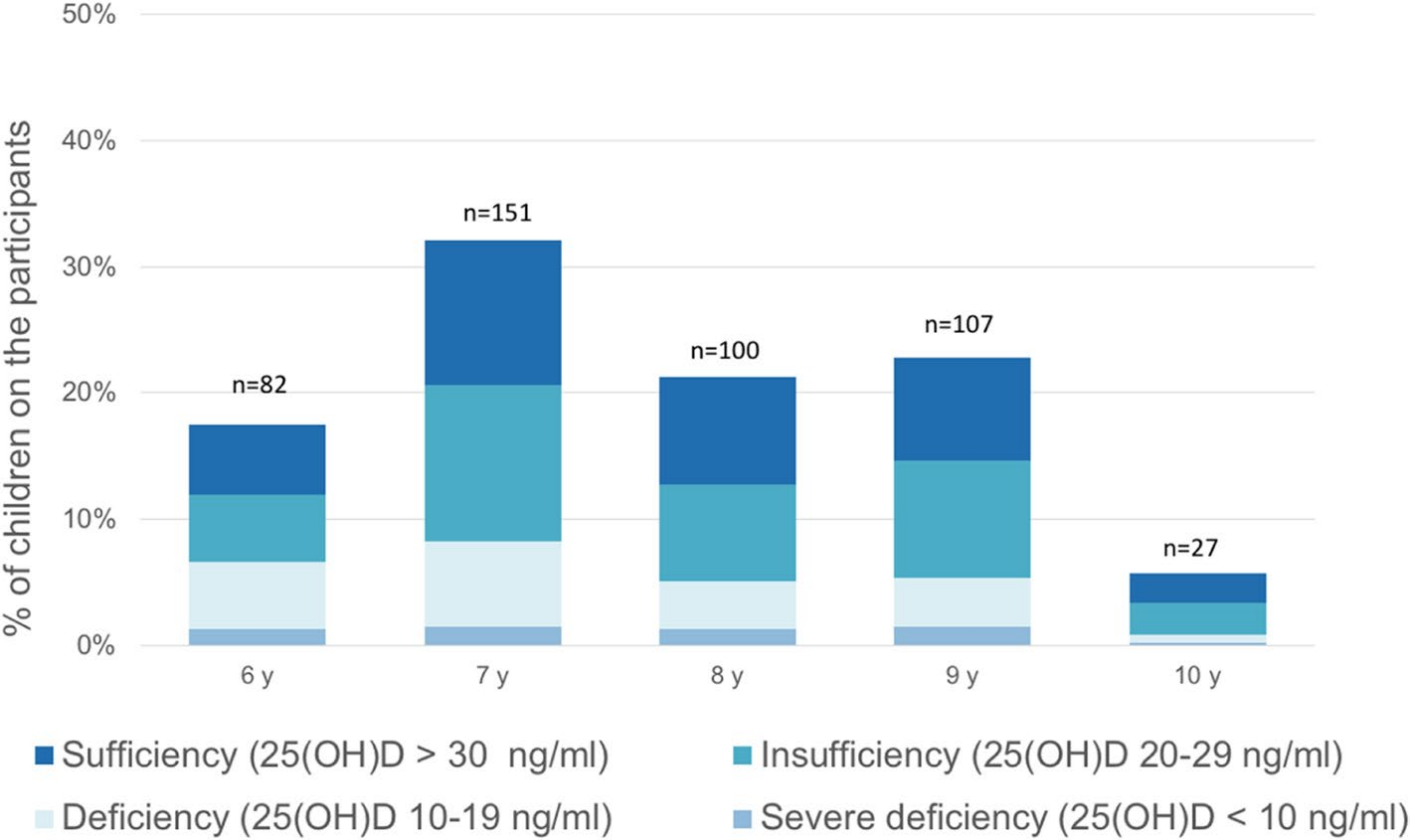
Percentage of children by serum 25-hydroxyvitamin D [25(OH)D] level for age groups. The labels above each column refer to the number of children in each age category. The age-range goes from 6 to 10 years. As expected for such a small age range, there are no significant differences on the distribution of 25(OH)D levels compared by age

Children with BMI > 95 percentile had significant higher prevalence of 25(OH)D deficiency (25(OH)D < 20 ng/mL) compared to their normal counterparts with BMI 5- 85 percentile (*p* = 0.036) (Fig. 3). The distribution of 25(OH)D levels according to gender and age showed no significant statistically differences (Fig. 4), (Fig. 5).

In a multiple regression analysis vitamin D status was predicted by season of blood sampling (*p* < 0.0001), ethnicity (*p* < 0.0001) and BMI percentiles groups (*p* = 0.017), (R^2^ = 0.464).

**Fig. 6:**
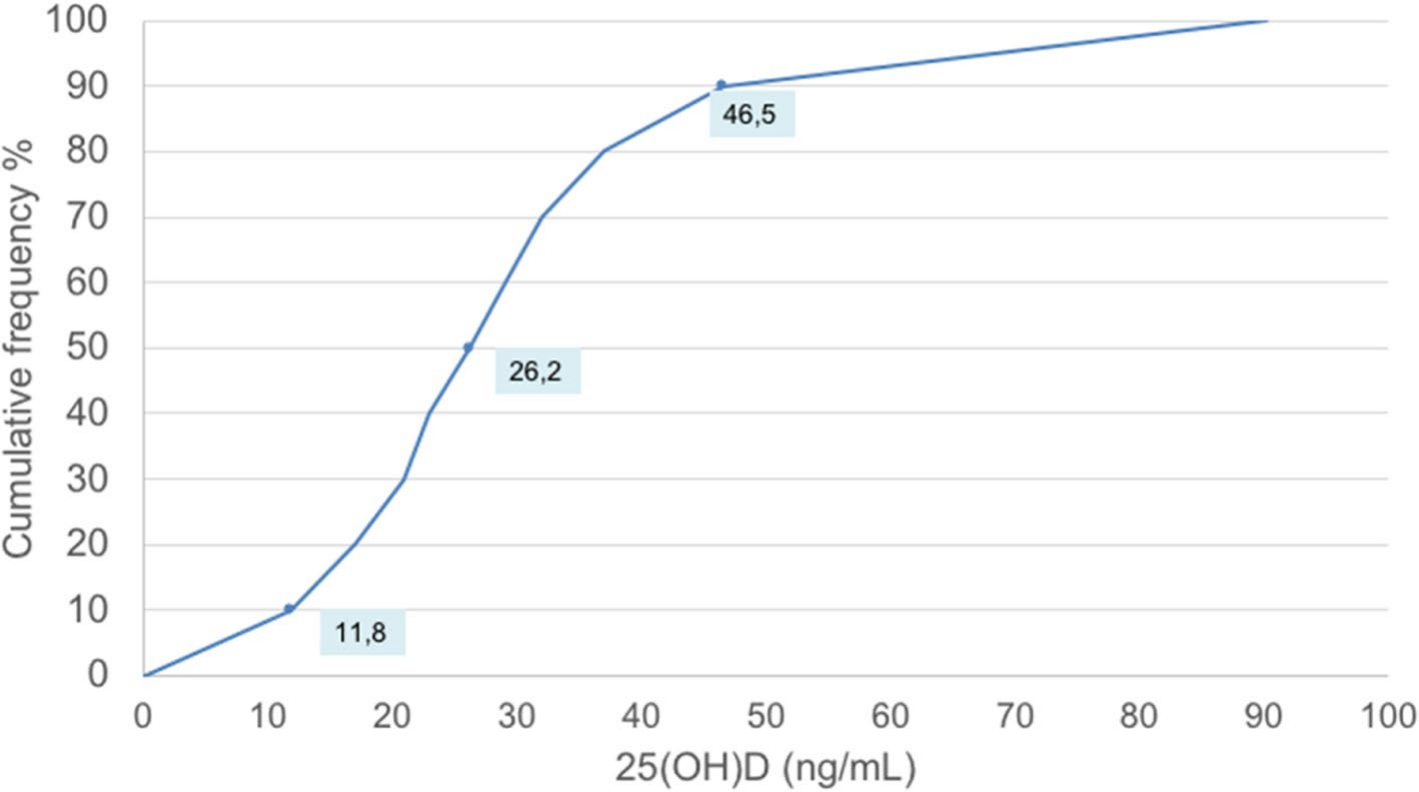
Cumulative frequency distribution of serum 25-hydroxyvitamin D [25(OH)D] concentration in the study children. The labelled values refer to 25(OH)D value at the 10,50,90th percentile

Vitamin D concentration was also analyzed in order to obtain a distribution of percentiles (Fig. 6). The cumulative frequency distribution of serum 25(OH)D in the study children was also studied (Fig. 6).

## Discussion

Several observational and cross-sectional studies on vitamin D in children and adolescents in Europe are characterized by high variability among the different countries and by relatively high prevalence rates of deficiency and insufficiency, explained in part by the different cut-off values used and by the different laboratory assays performed. Furthermore most of these studies included selected groups of children or adolescents recruited in the hospital setting [21, 22, 25] and/or healthy adults [26–29]. This cross-sectional study including a representative sample of school-age children from an urban area in Italy clearly shows a high prevalence of 25(OH)D insufficiency in this age-group, with 63.8% of children with 25(OH)D level below the desirable threshold of 30 ng/mL, and a deficiency (25(OH)D < 20 ng/mL) in 26,6% of children during at least some part of the year. These results reflect the situation of the general pediatric Italian population, since the study children were enrolled in schools and not in clinical settings, without any selection or filtering process. These findings are consistent with previous studies conducted in children from other areas of Italy and Europe [21, 22, 30]. A systematic review showed that, despite relative abundance of sunlight availability in the south Europe and east Mediterranean area, the average prevalence of circulating 25(OH)D concentration below 10 ng/mL ranged from 4 to 18%, while 25(OH)D concentration below 20 ng/mL ranged from 35 to 75% [31]. A study conducted among schoolchildren in Greece reported that the prevalence of 25(OH)D concentration < 12 ng/mL and < 20 ng/mL was 5,2% and 52,5%, respectively [32].

The situation is not different in other latitudes. A recent study conducted in Polish children aged 9–13 years found a vitamin D deficiency in the majority of them (64%), particularly after the winter period [33]. In the United Kingdom 35.1% of children and adolescents were vitamin D insufficient (25(OH)D concentration below < 20 ng/ml), while among Danish children this deficiency status was found in 28.4% of them, and in one-fifth of Finnish Children [34–38]. In the UK population serum vitamin D levels were strongly associated with ethnic group. Mean levels were double for white children (26,04 ng/ml) compared with non-white children (12,82 ng/ml) [34].

A study conducted among native Dutch and first- and second-generation non-Western immigrants showed that half of the pediatric population examined had serum 25(OH)D levels below 20 ng/mL, with Non-Western immigrants at increased risk for vitamin D deficiency compared to their native Dutch peers [37]. Although our study population was mainly of Caucasian ethnicity (90%), we found an increased risk of vitamin D deficiency in non-Caucasian children. The association between ethnicity and low vitamin D levels was confirmed in another Italian study showing that children of African, North African and Indian descent had higher prevalence of vitamin D deficiency than their white counterparts [22]. As expected, season is a strong determinant of serum vitamin D level, with winter and spring months representing the period with the highest prevalence of vitamin D deficiency and insufficiency, as also reported in other studies [22, 32, 33, 38].

We found a significant vitamin D deficiency in obese as compared to normal weight children (mean 25(OH)D level: 23.6 vs. 29.1 ng/mL, *p* = 0.023). The potential harmful association between obesity and risk of vitamin D deficiency among children is well known [39]. A possible explanation is that since vitamin D is fat soluble and likely sequestered in lipid droplets of adipocytes when stored, in individuals with obesity it may lead to low levels of vitamin D in blood despite the large amounts of vitamin D located in adipose tissue. Due to changes in lifestyle, some authors even refer to the convergence of two epidemics: obesity and vitamin D deficiency [40]. A study conducted in southeastern United States among adolescents, found a significant inverse correlation between 25-hydroxyvitamin D level and all adiposity measurements, i.e. BMI percentile, waist circumference, total fat mass, percentage of body fat, visceral adipose tissue, and subcutaneous abdominal adipose tissue [41]. These data are also confirmed by other studies [34, 42]. In the present study, no significant differences in 25(OH)D levels were observed between males and females. These data are in contrast with other reports [30, 32], that showed lower 25(OH)D levels in females. Moreover, because all participants lived in urban area, it was not possible to establish if vitamin D may be influenced by the living area (urban or rural).

Other potentially influencing factors could be eating habits, lifestyle, minutes of daily sun exposure, use of sunscreens but they were not investigated in this study.

The main limitation of the study was the relatively small sample size. Nevertheless, we would like to point out as a strength that the present study investigated healthy school-age children enrolled in schools and not in clinical settings with a well-defined age-range.

Furthermore, in Italy vitamin D fortification of food is not common as in other countries [38], an observation that reduces a source of uncontrolled variation. Finally, the blood sampling took place throughout the year, so blood samplings captured the full range of seasonal variations. Almost all children were sufficient, even by Italian standards, and while a few were insufficient, none was deficient in summer. This is a reassuring message, even if for the rest of the year the rate of deficiency (as defined by the Italian Society for Preventive Pediatrics) is quite high [43–46].

## Conclusions

In conclusion, despite the southern latitude, our study shows that the majority of Italian schoolchildren have vitamin D deficiency (25(OH)D < 20 ng/ml), particularly during winter and spring time, in agreement with the prevalence reported in other European pediatric population. The most powerful determinants of vitamin D deficiency are obesity, the ethnicity and the season. In terms of prevention, it is important to encourage children to implement their leisure time in outdoor physical activity and daily sun exposure, paying attention to the need for vitamin D supplementation during winter.

## Data Availability

All data generated or analyzed during this study are included in this published article.

## Abbreviations

1,25(OH)D: 1,25-Dihydroxyvitamin D
25(OH)D: 25-Hydroxyvitamin D
NORDEN: Nordic council of ministers
SACN: UK Scientific advisory committee on nutrition
EFSA: European food safety authority
ESPGHAN: European society for paediatric gastroenterology hepatology and nutrition
SIPPS: Italian pediatric society and the italian society of preventive and social pediatrics
BMI: Body mass index
Ca: Calcium
P: Phosphate
SD: Standard deviation
